# Special Issue “Molecular and Genetic Aspects of SARS-CoV-2 Infection and COVID-19 Disease”

**DOI:** 10.3390/ijms25094670

**Published:** 2024-04-25

**Authors:** Fernando Cardona, Jordi Pérez-Tur

**Affiliations:** 1 Unitat de Genètica Molecular, Institut de Biomedicina de València-CSIC, Jaume Roig 11, 46010 València, Spain; 2 CIBERNED, ISCIII, 46010 València, Spain

We are pleased to present the first and second editions of this Special Issue, titled “Molecular and Genetic Aspects of SARS-CoV-2 Infection and COVID-19 Disease”, of the *International Journal of Molecular Sciences*.

More than four years after the onset of the SARS-CoV-2 pandemic, an impressive amount of knowledge has been gathered. This Special Issue aims to provide a summary of molecular knowledge about the virus, as well as the mechanisms underlying the infection, host response, effects on pathology, evolution of the virus and pandemic, and recommended therapeutic strategies.

During SARS-CoV-2 infection, the viral spike protein, and, in particular, the receptor-binding domain (RBD), interacts with the host ACE2 receptor, facilitated by TMPRSS2 protease activity [1]. Regarding vaccine development, mRNA protein subunit-based vaccines have demonstrated efficacy in eliciting protective immune responses against SARS-CoV-2 [2]. Structural studies elucidate the vaccine-antibody–antigen-receptor interactions critical for optimizing vaccine design, as well as those that enhance therapeutic efficacy and safety profiles [3]. The interactions between the human ACE2 receptor and CBD may differ between viral variants [4]. In this Special Issue, Hognon et al. [5] explain the differential infectivity of viral variants by the differences in their interaction with ACE2.

Host genetics and gene expression regulation have been shown to be key factors in the response to the infection [6]. Examples include interferon genetics and regulation [7], or levels of the human receptor ACE2 and the protease TMPRSS2 [8].

Diagnostic advances are all based on molecular techniques. PCR-based techniques, rapid antigen tests, CRISPR-based diagnostics, and next-generation sequencing technologies have revolutionized COVID-19 diagnostics and have enabled the rapid and accurate detection of viral infections, as well as the monitoring of viral variants [9,10,11,12].

Some studies suggest the involvement of heparan sulfate proteoglycans to facilitate this entry, such as Syndecans [13]. However, it is still unclear how other host genetic factors or viral variants affect their interaction and the entry of the virus into the cell. Several genetic association studies link a locus on chromosome 3 with COVID-19 severity; this locus harbors multiple genes—among them, CCR5 [14,15]. In this Special Issue, Cantalupo et al. [16] investigate the role of regulatory noncoding and predicted pathogenic coding variants of CCR5 in predisposing individuals to severe COVID-19. The authors demonstrate that these variants are associated with an increased risk of severe disease. CCR5 encodes a chemokine receptor and is expressed by immune system cells such as T cells [17,18]. Two main effector cell types are involved in the anti-viral response, NK and T cells. The response and the activation of NK cells comprise the first-line defense against viruses, and their failure activates adaptive cellular response [19]. Immune dysregulation underpins COVID-19 pathogenesis, characterized by cytokine storms, lymphopenia, and impaired adaptive immunity [20,21]. Jassem et al. [22] present a case report of a patient with persistent SARS-CoV-2 infection who was successfully treated with activated cytotoxic T and NK cells. This approach may be useful in the treatment of patients with persistent infection. Post-acute sequelae of SARS-CoV-2 infection, known as long COVID, present diverse clinical manifestations, including fatigue, cognitive impairment, and organ dysfunction [23]. Molecular, cellular, and immune studies elucidate underlying mechanisms and inform targeted interventions [24].

Additionally, it is now clear that SARS-CoV-2 variants play a significant role in the extent of infection and the spread of the virus, and these may also impact the immune response, which is either acquired or induced by vaccination [25,26]. Insights into immune evasion mechanisms facilitate the development of targeted immunotherapies. In addition, viral variants are also an important factor in determining infection and disease severity. In this Special Issue, Hudak et al. [27], explore the role of Syndecan-4 in the transmission of the SARS-CoV-2 Delta variant. The authors demonstrate that Syndecan-4 facilitates entry into cells of the Delta variants and enhances its ability to spread. This Special Issue also published two review articles providing an explanation of the virus variants and different transmissibility, as well as the virus’s ability to escape immune protection induced by vaccination and previous infection [28,29]. The authors discuss the characteristics of this variant and its potential for causing a “silent epidemic”, as well as an overview of the genetic and clinical peculiarities of the Omicron variant and its potential to overturn the SARS-CoV-2 pandemic.

All this knowledge should be applied in the design of therapeutic strategies, utilizing both pharmacologic and epidemiologic interventions. The main risk group for severe disease is the elderly, but the mechanisms by which this occurs are not fully understood. In addition to patients of advanced age, those who are obese, immunocompromised, and receiving chemotherapy for cancer have been classified as vulnerable groups for COVID-19 [30,31]. In this regard, Tyrosine Kinase inhibitors are widely used in cancer treatment [32], and some of them, such as Imatinib, have been proposed to treat both diseases simultaneously—cancer and COVID-19 [33]. In this Special Issue, Lin et al. [34] investigate the use of Imatinib, a drug used to treat leukemia, as a potential therapy for COVID-19. The authors demonstrate that Imatinib inhibits the expression of ACE2, which is required for the virus to enter cells.

Overall, this Special issue provides a comprehensive overview of the current understanding of SARS-CoV-2 and COVID-19. We hope that these articles will contribute to ongoing efforts to combat the pandemic and improve our understanding of the virus.
